# Silent and dangerous: catheter-associated right atrial thrombus (CRAT) in children on chronic haemodialysis

**DOI:** 10.1007/s00467-020-04743-9

**Published:** 2020-10-30

**Authors:** Martin Garcia-Nicoletti, Manish D. Sinha, Alexandra Savis, Shazia Adalat, Narayan Karunanithy, Francis Calder

**Affiliations:** 1 Department of Paediatric Nephrology, Evelina London Children’s Hospital, London, SE1 7EH UK; 2 Kings College London, London, UK; 3 Department of Paediatric Cardiology, Evelina London Children’s Hospital, London, UK; 4 Department of Intervention Radiology, Evelina London Children’s Hospital, London, UK; 5 School of Biomedical Engineering & Imaging Sciences, King’s College London, London, UK; 6 Department of Paediatric Transplantation, Evelina London Children’s Hospital, London, UK

**Keywords:** CRAT, Catheter-associated right atrial thrombi, Right atrium, Thrombus, CVC, Tunnelled, Fistula, Arteriovenous, KFRT, Children

## Abstract

**Background:**

Catheter-associated right atrial thrombus (CRAT) is a recognised complication of central venous catheter (CVC) use for haemodialysis (HD) patients.

**Methods:**

This was a single-centre retrospective longitudinal observational study of consecutive children aged 6 months–18 years over a 7-year period receiving in-centre chronic HD. Echocardiograms as per routine cardiac surveillance were performed 6 months or earlier given clinical concerns.

**Results:**

Sixty-five children, 36 boys (55.4%), median (IQR) age 11.8 (5.3, 14.7) years, received HD for kidney failure with replacement therapy (KFRT). Initial modality was HD in 45 (69.2%), with CVC as initial access in 42 (93.3%) and AVF in 3 (6.7%); in the remaining 20 (30.8%) patients PD was the initial modality before switching to HD. Seven of 65 (10.8%) developed CRAT at median 2 (0.8, 8.4) months from CVC insertion, with one CRAT detected 3 days following insertion. One child had 2 episodes of CRAT and one additionally thrombosed their AVF. No patient had an underlying primary kidney disease associated with a pro-thrombotic state. Those with CRAT were younger, had more frequent CVC change and received dialysis for longer duration compared to those with no CRAT. Six episodes of CRAT (75%) received anticoagulation therapy. Infective complications were observed in 25% and catheter malfunction in 50%. Five CRAT episodes (62.5%) resulted in CVC loss. One patient died after a haemorrhagic complication of anticoagulation and sepsis, and another developed life-threatening superior vena cava obstruction syndrome. Overall mortality 14% (1/7).

**Conclusions:**

This is the first report of CRAT in a paediatric HD population. There was ~ 11% incidence of CRAT in patients receiving chronic HD detected by surveillance echocardiography. Although frequently asymptomatic, CRAT is associated with serious sequelae. Anticoagulation and surveillance with expert echocardiography remain mainstays of management.

Graphical abstract
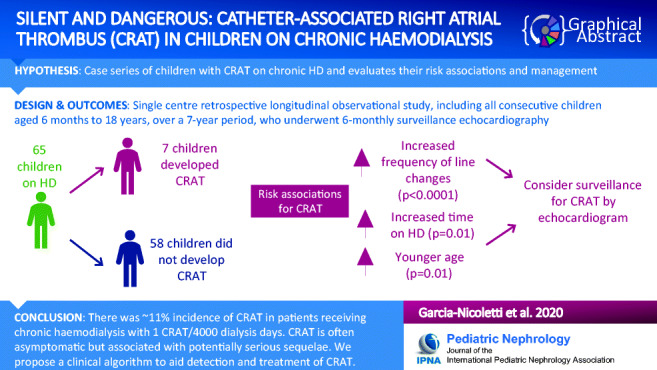

**Electronic supplementary material:**

The online version of this article (10.1007/s00467-020-04743-9) contains supplementary material, which is available to authorized users.

## Introduction

Haemodialysis (HD) via a central venous catheter (CVC) for children with kidney failure with replacement therapy (KFRT) remains the commonest form of dialysis in childhood [1]. For young children aged < 2 years, peritoneal dialysis (PD) remains the recommended dialysis access of choice [2], as this modality is associated with fewer complications [1]. If the duration of dialysis is estimated to be 'short-term', for example less than 6 months before a planned kidney transplant, dialysis via a CVC may be adequate to access the vascular system. Despite the expected 'short-term' period, children often remain on HD for extended periods of time. The International Pediatric Hemodialysis Network Registry recently reported that those receiving HD via a CVC and awaiting transplantation waited a median of 14 months, with one-quarter of patients remaining on dialysis therapy for more than 3 years [3]. However, for longer term HD, consideration should be given to form an arteriovenous fistula (AVF), as long-term HD via a CVC carries significant risks including infection, malfunction, inadequate dialysis, hospitalisation, and central venous stenosis [4]. Despite the known lower complication rates associated with AVF versus CVC [1], European and US databases highlight that the vast majority of children on chronic HD continue to receive dialysis via a CVC [5, 6]. Reasons for this may include lack of surgical expertise, clinician preference, and patient and family acceptance of an AVF.

Catheter-associated right atrial thrombus (CRAT) is a potentially serious complication related to the use of CVCs and has been previously described in adults on HD with significant sequelae including pulmonary embolism, infection, septic emboli and associated mortality rates of 18–27% [7, 8]. CRAT is likely to be an under diagnosed complication of CVC use as there is currently no standard definition. CRAT is commonly asymptomatic and revealed only after an associated complication. It can be difficult to diagnose and requires specialist echocardiographic assessment [9, 10].

In this report, we present a case series of seven children on HD diagnosed with CRAT, discuss their management and suggest an algorithm for surveillance in children with CVCs on chronic HD.

## Methods

This was a single-centre retrospective longitudinal observational study, including all consecutive children aged 6 months to 18 years, over a 7-year period (2013–2019) receiving in-centre chronic intermittent HD at Evelina London Children’s Hospital, UK.

We collected data including demographics, age at start of dialysis, modality at start of kidney replacement therapy (KRT) and time on dialysis before initial echocardiogram study as well as duration between previous echocardiogram study and detection of CRAT. Ethnicity was defined as stated by the patient/family and recorded on the electronic patient records.

### Inclusion and exclusion criteria

Children were included in the study if the following were present including: (i) received HD for ≥ 3 months and (ii) one or more M-mode 2D echocardiogram studies available. Children were excluded from the study if HD was for < 3 months (e.g. acute kidney injury). We additionally excluded all children who received peritoneal dialysis only for KRT during the course of the study.

### Pro-thrombotic work-up

As part of our centre protocol, baseline coagulation tests are performed in children prior to insertion of any CVC, which include an INR and prothrombin time. Detailed clinical history for any personal and family history of thromboembolic events was taken. All children additionally underwent further tests following commencement of HD to identify any pro-thrombotic conditions as part of their pre-transplantation evaluation. Investigations for inherited and acquired abnormalities of coagulation including anticardiolipin antibodies (IgM and IgG), lupus anticoagulant, protein S, protein C, antithrombin III, factor V and screening for prothrombin 2010 mutation were performed.

### Echocardiogram studies

As part of routine surveillance, echocardiograms are performed at 6-month intervals or earlier if there are any clinical concerns. All patients included in this case series were diagnosed with CRAT following identification on echocardiography performed by experienced paediatric cardiac sonographers. Data reported in this study include findings on serial echocardiography (including follow-up post kidney transplantation) until resolution of CRAT or the time of their last available follow-up. The echocardiogram study performed nearest to commencement of HD was termed as the ‘baseline’ study. A single senior paediatric cardiac sonographer (author AS) analysed all echocardiogram studies and the findings confirmed following review with a paediatric cardiologist.

### Statistical analysis

Subject characteristics are summarized as means ± standard deviation (SD) or median and interquartile range (IQR) if not normally distributed. Analysis was performed using SPSS version 25 (SPSS Inc., Chicago, Illinois) and *P* < 0.05 was taken as significant.

## Results

Sixty-five children, including 36 boys (55.4%) of median (IQR) age 11.8 (5.3, 14.7) years, received intermittent HD for established KFRT. Initial dialysis modality for KRT was HD in 45 (69.2%) children. Of those on HD, CVC was the initial dialysis access in *n* = 42 (93.3%) and AVF in *n* = 3 (6.7%) patients. In the remaining 20 (30.8%) children, dialysis was commenced via PD catheter initially before subsequently changing to HD following PD-related complications. Table 1 provides additional details regarding patient characteristics and data regarding CVC site.

**Table 1:** Demographic and clinical characteristics of *n* = 65 children who received chronic intermittent haemodialysis

	No CRAT	CRAT
Age at initial CVC insertion median (IQR), years*	10.9 (5.8, 15.3)	5 (0.9, 10.3)
Median number of CVC per patient^	1	4
Duration on haemodialysis median (IQR), years*	1.2 (0.4, 1.7)	2.5 (1, 3.8)
Sex		
Male, *n* (%)	32 (55%)	4 (57%)
Female, *n* (%)	26 (45%)	3 (43%)
Primary kidney disease		
CAKUT, *n* (%)	15 (26%)	4 (57%)
Glomerular, *n* (%)	23 (40%)	2 (29%)
Other, *n* (%)	20 (34%)	1 (14%)
Site of first CVC		
RIJV	48	6
LIJV	2	1
Unknown	8	0
Outcome at the end of study follow-up		
Transplantation	39 (67%)	6 (86%)
Continues on HD	7 (12%)	0 (0%)
Continues on PD	1 (2%)	0 (0%)
Care transferred to adults or other centre	6 (10%)	0 (0%)
Death	5 (9%)	1 (14%)

*CVC*, central venous catheter; *P = 0.01; ^*P* < 0.0001; *CAKUT*, congenital anomaly of kidney and urinary tract; *RIJV*, right internal jugular vein; *LIJV*, left internal jugular vein; *HD*, haemodialysis; *PD*, peritoneal dialysis

### CRAT identification

Fifty-nine children (90.8%) had an initial echocardiogram study at median (IQR) of 3 (0.7, 6.9) months following commencement of HD. Seven of 65 (10.8%) children developed CRAT (four boys, three girls) (Table 2) in the study period. Patients with CRAT received HD for median (IQR) of 2.5 (1, 3.8) years versus those with no CRAT who received HD for 1.2 (0.4, 1.7) years (*P* = 0.01). In all patients receiving HD, the incidence of CRAT was found to be 1/4000 CVC days.

**Table 2:** Demographics and clinical characteristics of seven children with catheter-related atrial thrombus (CRAT) over a 7-year period during which *n* = 65 children received chronic intermittent haemodialysis

Case	Age at time of CRAT Gender	Weight at time of CRAT detection (kg)	Primary kidney disease	CVC lines before CRAT (n)	CRAT detection after CVC insertion	Concurrent line infection?	Anticoagulation (days)	Anticoagulation medications	Outcome A: CRAT risk B: dialysis access C: outcome /CRAT resolution
1	6 years^1^ Female	1st CRAT: 16.4 kg	Unknown	3	20.1 months	1st no	1st 0	1st CRAT managed conservatively	1st CRAT A: low-moderate risk, affecting flow B: CVC was removed due to persistent febrile episodes (no organism was identified) C: 1st thrombus resolution on echo after 20 days
		2nd CRAT: 15.9 kg				2nd no	2nd 31.2 months (ongoing until end of study period)	2nd tissue plasminogen activator, intravenous (IV) heparin and warfarin	2nd CRAT A: high risk B: reverted back to HD via new CVC due to being unable to initiate on PD. Subsequently reverted back to PD due to SVC obstruction (bilateral central neck vein stenosis-occluded internal jugular veins bilaterally and an occluded right subclavian vein) before being reverted back to HD due to peritonitis. Transplanted 6.4 months after recommencing HD C: thrombus resolution on echo end June 2019 and continues on warfarin.
2	3 years Male	17.04 kg	Focal segmental glomerulosclerosis	6	3 days	Yes ^2^	4.1 months	IV Heparin followed by warfarin	A: High risk, concurrent infection B: Converted to PD 1 month after anticoagulation ceased and CVC removed. Transplanted 2 months following cessation of warfarin. C: Resolution of thrombus at 4.1 months.
3	4 years Male	14.7 kg	Posterior urethral valves	3	23 days	Yes^3^	1.4 months	IV heparin and warfarin	A: high risk. Patent foramen ovale with intermittent left to right flow, concurrent infection B: converted to PD, CVC removed C: deceased due to large right fronto-parietal acute parenchymal haemorrhage
4	15 years Male	50.3 kg	VACTERL	2	29 days	No	20 days	IV heparin and warfarin	A: low risk B: formation of AVF 3 months following identification of CRAT; CVC removed once AVF matured and being used. Transplanted 18 months following diagnosis of CRAT. C: stable appearance of ‘low risk’ thrombus on serial ECHOs therefore anticoagulation discontinued after 20 days. Resolution of thrombus at echo 10 months later but continued on low dose aspirin.
5	10 years Female	40.9 kg	Dysplasia	1	2.3 months	No	4.1 months	IV heparin and warfarin	A: high risk B: continued to dialyse via same CVC until transplanted. Anticoagulation ceased following transplant and CVC removal. C: resolution of thrombus on echo at 3 months
6	4 years Female	9.9 kg	Bilateral Wilms	3	8.4 months	No	4.7 months	IV heparin and warfarin	A: moderate risk B: CVC removed. Converted to PD. 6 months following CRAT, AVF that had been created failed as AVF thrombosed. C: CVC line removed after 5 months and anticoagulation discontinued. Resolution of thrombus at 15 months
7	4 years Male	16.2 kg	Posterior urethral valves	4	2.0 months	No	0 days	N/A (managed conservatively)	A: low risk B: continuing to dialyse via same CVC with no apparent sequelae until transplanted C: resolution of CRAT on repeat echo at 7 months

^1^Age at first CRAT diagnosis

^2^*Candida* and *Staphylococcus aureus*

^3^*Staphylococcus aureus*

During the study period, there were 109 CVC insertions: in 7 patients with a CRAT 29 CVCs were placed and 80 CVCs in 58 patients without a CRAT. Only 1 of 7 patients with CRAT had no line change compared with 36 of 58 patients with no CRAT (*P* = 0.02).

In those with CRAT, the median (IQR) time from insertion of CVC to detection of CRAT was 2 months (0.8, 8.4), with one CRAT detected 3 days following insertion of CVC. Six of the seven patients (87%) with CRAT had multiple CVCs previously with a median (IQR) of 4 (2, 5). At the time of CRAT, their median (range) age was 4 (3–15) years and median (range) weight 16.4 (9.9–50.3) kg.

All CVCs were inserted under general anaesthesia by a consultant interventional radiologist in a dedicated catheter lab suite using ultrasound and fluoroscopic guidance. The final position verified using on table angiographic screening with the line tip positioned in right atrium.

Two children had recurrent thrombi despite a normal thrombophilia screen. One child (case 1, Table 2) was observed to have two distinct episodes of CRAT. She had presented with rapid deterioration of her kidney function and on kidney biopsy was found to have granulomatous tubulointerstitial nephritis (TIN) of unknown cause. Clinically, an underlying vasculitis was suspected (persistently high ESR, hyperferritinaemia, multiple small volume lymph nodes on whole body MRI, anaemia and lymphopenia, splenomegaly, hepatomegaly and prolonged unexplained febrile episodes associated with intermittent livedo rashes). Extensive investigations failed to identify cause of the granulomatous TIN or a possible vasculitis. Another child had a CRAT and subsequently also developed a thrombus in her AVF (Table 2).

### Pro-thrombotic status

We were unable to identify a specific pro-thrombotic condition in the child suspected to have vasculitis (case 1, Table 2). None of the other patients had an underlying primary kidney disease associated with a pro-thrombotic state as assessed by blood tests looking for inherited and acquired abnormalities of coagulation or by interrogating for personal and family history of thromboembolic events. The patient with FSGS (case 2, Table 1) was anuric on HD with normal plasma albumin at 48 g/L at the time of diagnosis of CRAT. Another child with CRAT was found to have lupus anticoagulant but with no prior thrombotic episodes. On echocardiogram, no patient had severe reduction in cardiac contractility that could have favoured the occurrence of CRAT.

### Management of CRAT

**Fig. 1:**
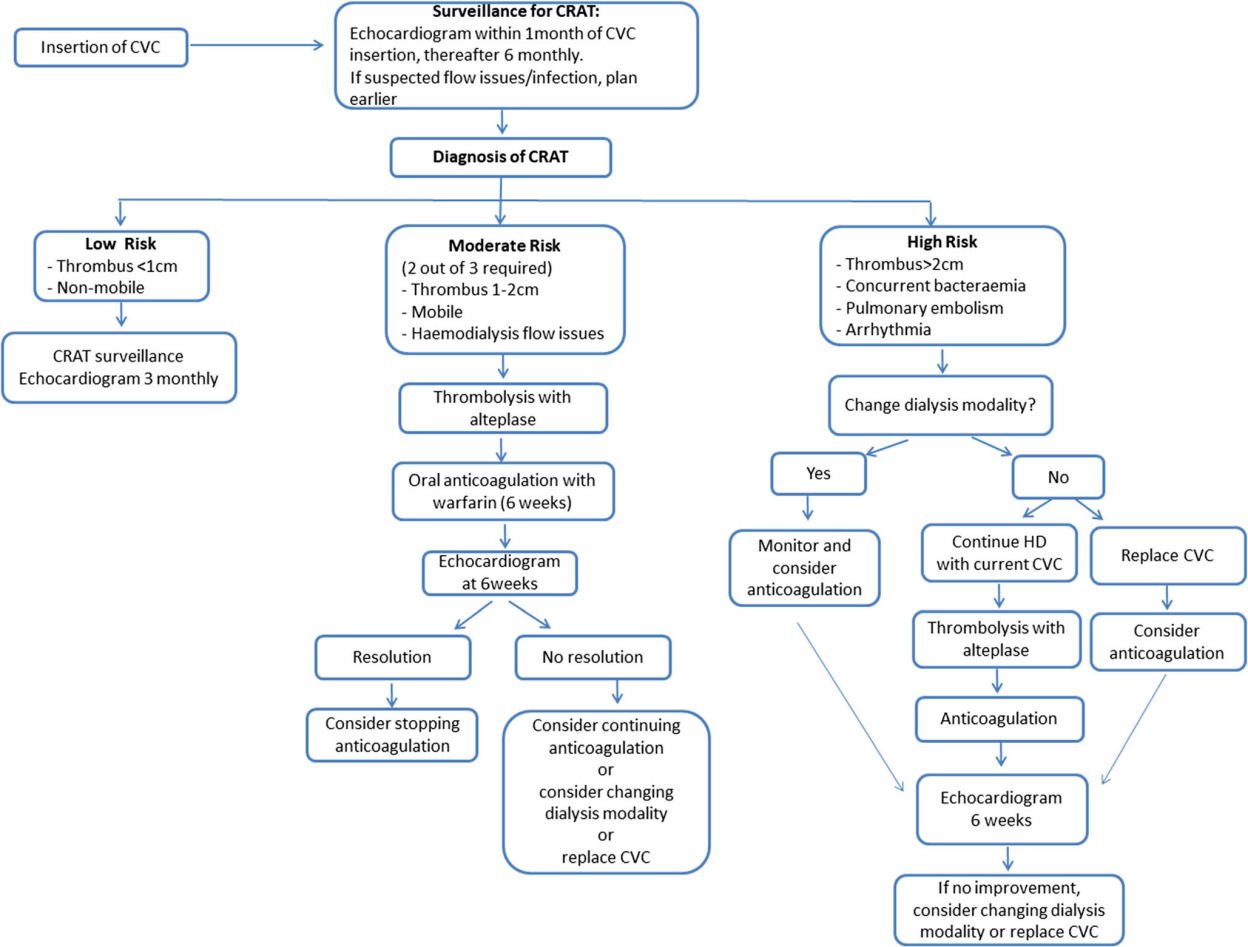
Clinical algorithm for the clinical management of children with catheter-related atrial thrombus (CRAT)

Following initial diagnosis, serial echocardiography was used for assessment and monitoring of CRAT in all children with median (range) of 5 (3–7) echocardiogram studies per patient. Two of 8 cases (25%) with CRAT were not anticoagulated following discussion at our multidisciplinary review, as these thrombi were considered to be clinically insignificant based on both echocardiographic appearances (small < 1 cm, non-mobile, located at catheter tip only), absence of haemodynamic effects (no observable effect on blood flows on dialysis) and with no other clinical sequelae (see Fig. 1 classification). Both patients had spontaneous resolution of their CRAT without treatment and continued to receive dialysis without removal of CVC or change of access. One of these children after a period on PD, complicated by peritonitis, had to be re-commenced on HD. Soon after the second CVC was re-inserted, the patient developed another CRAT with clinically severe superior vena cava obstruction (SVCO) and secondary chylous pleural effusions. Imaging demonstrated thrombosis of the SVC and both brachiocephalic veins. This was eventually  successfully treated with catheter directed thrombolysis and SVC stenting after a period of intensive respiratory therapy. The child went on to have a successful kidney transplant.

In all cases, where possible, an attempt was made to preserve a functioning CVC. Treatment with anticoagulation was used in moderate and high-risk cases with careful monitoring of CRAT for progression or complications. Six of 8 episodes of CRAT (75%) received anticoagulation therapy. All cases that were anticoagulated were commenced on intravenous heparin infusions and then converted to warfarin, aiming for a target INR of 2–3. The duration of warfarin treatment median (range) was 4.1 (20 days—4.7 m) months. Anticoagulation was discontinued following resolution of CRAT on serial echocardiogram studies.

Five of 8 episodes of CRAT (62.5%) resulted in removal of CVC. Two patients had their CVC removed due to associated catheter-related bacteraemia. One child had their CVC removed after repeated pyrexia of unknown cause. One CVC was removed following superior vena cava obstruction syndrome. Another patient had their CVC removed following a persistent CRAT despite anticoagulation therapy.

### CRAT complications

#### Infection

—2 of 7 (28.6%) patients with CRAT had bacteraemia versus 11 of 58 (18.9%) patients with no CRAT (*P* = 0.62) in the study period. Two children had confirmed bacteraemia on blood culture (1 *Staphylococcus aureus* bacteraemia and 1 mixed *Staphylococcus aureus* and *Candida* bacteraemia). Another patient had their CVC removed following recurrent febrile episodes although no organism was identified on repeated blood culture.

#### Catheter malfunction

—occurred in 4 of 8 CRAT episodes (50%). These were reported as either targeted flow rates not being achieved or lines described as ‘sucking’ or being ‘stiff’ during dialysis and requiring recurrent use of fibrinolytic agents to improve the CVC flow rates; data regarding catheter malfunction in those with no CRAT were not systematically recorded.

#### Respiratory complications

—secondary to a severe SVCO syndrome as described above.

### CRAT outcome

Seven of 8 (87.5%) CRAT showed complete resolution of thrombus on serial echocardiogram studies. Two patients experienced complications relating to anticoagulation therapy, one bled into their peritoneal dialysate and another had spontaneous bleeding from a previous femoral arterial catheter site. One child with a CRAT developed *Staphylococcus* sepsis and septic emboli including a cerebral abscess. The patient developed a large right fronto-parietal acute parenchymal haemorrhage as a complication of anticoagulation. He died following blockage of urgently placed intraventricular shunt.

## Discussion

This is the first case series of CRAT in a dedicated paediatric dialysis unit, including novel data regarding incidence of CRAT in this high-risk population, based on a surveillance programme of regular echocardiogram studies. We report that although CRAT is often asymptomatic, it is not a benign finding and highlights the risks of HD via a CVC in all age groups in childhood. Pre-emptive transplantation, use of PD and promotion of AVF formation for HD are fundamental to avoid this potentially fatal condition.

### Diagnosis

There are no reports regarding incidence or prevalence of CRAT in the paediatric dialysis literature. The reported incidence of CRAT in the adult dialysis population is variable (2–29%) [9]. However, diagnosis is impaired by the absence of a precise definition. In this series, the diagnosis was made on routine echocardiography by detecting thrombus associated with a dialysis catheter and extending into the right atrium. In a non-dialysis paediatric population, Yang et al. classify CRAT as low risk (< 2 cm, immobile, linear) and high risk (> 2 cm, mobile, pedunculated) based on thrombus size, morphology and mobility on echocardiography [11]. Whilst useful in predicting CRAT-related embolic phenomena, this categorisation does not take into account several key issues relevant to the paediatric HD population, including the effect of CRAT on blood flow during HD, the inability to achieve adequate dialysis clearance, the natural history of a ‘low risk’ CRAT which may require surveillance without intervention, and complications related to anticoagulation and CVC replacement in a dialysis population.

The exact timing when a CRAT may initially form remains unknown since it is frequently asymptomatic. In this series, 6 of 8 cases of CRAT (75%) were identified incidentally following echocardiogram studies as part of screening for cardiovascular health in children on chronic HD with median (range) duration on HD at the time of diagnosis of CRAT at 20.1 (1.2–46.9) months. Taken together, these data highlight that CRAT can occur at any time in a child on HD.

In the adult literature, contrast venography is recommended for the diagnosis of CRAT [12]. To avoid radiation exposure, echocardiography by an experienced cardiac sonographer is the investigation of choice for children, but it is highly dependent on both the operator and the child cooperating [13]. ‘Poor flow’ on HD is a common manifestation of dialysis catheter malfunction, often related to a fibrin sheath. It is important to recognize that this may also be the first indication of a CRAT. In this series, 4 of 8 cases of CRAT (50%) of children had ‘flow problems’ reported by the dialysis staff and went on to have CRAT diagnosed by echocardiography. These data were not uniformly recorded in all children on HD but we have subsequently modified our practice, such that consistent poor flow issues are investigated by an echocardiogram within 4 weeks. It is noted however that the timing and frequency of echocardiogram is not stipulated via a standardised protocol, so there is likely variation across centres.

### Risk factors for development of CRAT

The factors predisposing to CRAT formation are not fully understood. However, the catheter itself is a major factor as it presents a pro-coagulant surface. The size of the catheter relative to the cavo-atrial junction especially in a paediatric population alters the blood flow and could contribute to CRAT and central vein stenosis. In this report, the children diagnosed with CRAT who were managed with removal of CVC did not develop further atrial thrombi as demonstrated on follow-up echocardiography surveillance studies.

In addition to the presence of the CVC, its position is a significant factor leading to CRAT formation. Ideally, the catheter tip should be placed in the right atrial chamber to achieve optimal flow rates during HD [14]. However, the CVC tip position is not static, for example, movement of the arms (during stretching) and thorax (during respiration) alters the position. As the tip moves up and down, it may contact the vessel or right atrial wall and damage the endothelial lining predisposing to thrombus formation [15]. Positioning the CVC tip above the right atrium may not achieve such good blood flows on dialysis but avoids the intra cardiac injury. However, such ‘tip injury’ in the superior vena cava is a significant factor leading to SVC stenosis [16] and may compromise future AVF options and increase the likelihood of SVCO syndrome.

A hypercoagulable state is also a significant risk factor for thrombosis. Coagulation in KFRT patients is complex—the aetiology of the primary kidney disease (vasculitis, nephrotic syndrome), intercurrent infection, hydration status and intra-dialytic hypotension may all vary and contribute to a pro-coagulant state. Many abnormalities can be detected by laboratory analysis, but their clinical significance remains poorly understood [17, 18]. In this series, only one child had any detectable abnormality (lupus anticoagulant positive) but no previous thrombotic events. A history of thrombosis with abnormal thrombotic screening would indicate the need for anticoagulation therapy for a child with a CVC [19]. The case for anticoagulation where there is no personal or family history and no inherited thrombophilia deficiency is a more complex risk-benefit analysis. Currently, there are no established guidelines for the use of anticoagulation in children with long-term catheter use for HD in the absence of coagulation abnormalities. A surveillance program monitoring for development of thrombi would seem a low-risk option rather than routine anticoagulation. If thrombi are detected, the CRAT should be risk stratified with consideration of anticoagulation and management of the CVC as per the suggested algorithm shown in Fig. 1.

In this series, 2 of 8 cases of CRAT (25%) experienced catheter-related infection. Whether CVC infection predisposes to CRAT or the CRAT was secondarily colonised by organisms remains unclear. However, suspicion of line infection or evidence of bacteraemia whilst on HD with a CVC should be investigated by an echocardiogram to exclude CRAT and any cardiac vegetations. If confirmed, consideration should be given to removing the CVC urgently.

### Treatment

The optimum treatment for CRAT is poorly defined, particularly in the paediatric population. A proposed algorithm by Stravroulopoulos et al. (2012) [9], following a meta-analysis that included 71 cases of CRAT in adult dialysis patients, suggested removal of the CVC or exchange over a guide-wire after therapeutic anticoagulation, to reduce the risk of pulmonary embolism. Antithrombosis guidelines for children published by Monagle et al. [19] recommend to anticoagulate if high risk (defined as > 2 cm in size and mobile) and consider thrombectomy or thrombolysis. However, if the thrombus is low risk, the advice is to consider removing the dialysis CVC with or without anticoagulation. The use of warfarin in adult patients with KFRT can be a problematic issue as it increases the risk of a major bleeding event, is known to accelerate vascular calcification, as well as being associated with and contributing to increased bleeding complications, hospitalisation and overall morbidity and altered pharmacokinetics [20, 21]. There are no robust data regarding warfarin use in children with KFRT [21]. In a recent small pilot study in children, high-risk patients, defined as those who recently had a CVC inserted for HD (defined as having active nephrotic syndrome, serum albumin < 25 g/L, urine protein creatinine ratio > 2 mg/mg or a previous CVC thrombus), were compared with patients with none of these risk factors [22]. The authors reported that treating high-risk patients with warfarin following insertion of their CVC had a median CVC survival of 369 days compared to 195 days in the control group. 83.3% of the high-risk group had a 1-year survival rate of their catheter compared to 16.7% in the standard group. Whilst this shows that warfarin is a possible option in extending the life of a CVC, there are no robust data reporting warfarin as a treatment option for children with CRAT on HD [22]. This study provides some information regarding this.

Removal of the dialysis CVC likely requires a temporary catheter insertion (in the neck or groin) and re-insertion of a permanent catheter at a later date. These interventions carry the potential for further complications such as infection, re-occurrence of CRAT and central venous stenosis that may impact on future transplant or AVF options. Anticoagulation alone carries a small but significant risk of significant haemorrhage. A systematic review of anticoagulation treatment in catheter-related thrombus by Kreuzinger et al. in an adult population showed duration of anticoagulation ranged from 8 days to 62 months [21]. There are no established guidelines on when to consider removal of CVC after CRAT identification.

We propose a treatment algorithm for the paediatric HD population, where the risk of CRAT is stratified into high, moderate and low. High risk is defined as a large (> 2 cm), hypermobile thrombus with serpiginous morphology or any sized CRAT with cardio-pulmonary complications (pulmonary emboli, SVCO, arrythmia) or associated bacteraemia (Fig. 1). Moderate risk includes thrombus (1–2 cm) extending from the CVC or affecting blood flows on HD. Low risk is an immobile thrombus (< 1 cm) attached to the catheter with no effect on HD or clinical sequelae. This algorithm is based upon retaining a functioning catheter (often with thrombolytic assistance), anticoagulation where necessary, and active surveillance of the CRAT for progression and/or complications.

In our case series, for the 2 low-risk cases, the CRAT resolved on follow-up at 7 and 10 months. One moderate-risk case showed resolution following conversion to PD and 4-month period of anticoagulation, the other moderate-risk case was converted to PD then reverted back to HD and developed a further CRAT and severe complications relating to SVCO syndrome and required long-term anticoagulation. Of the 4 high-risk cases, one child died from haemorrhage into an intracranial metastatic abscess whilst on anticoagulation. The other 3 cases resolved after a period of anticoagulation, one converted to PD, one continued dialysis via same CVC and one was unable to convert to PD and reverted back to HD via new CVC. If anticoagulation is contraindicated, the CVC should be removed and conversion to PD should be explored. If there is concurrent bacteraemia or complications arise while on anticoagulation, conversion to PD should also be considered. If PD is not feasible, urgent formation of an AVF should be considered for long-term dialysis access [6].

### Mortality

In this series of seven patients, there was one death (14% mortality). This 4-year-old boy was a high-risk patient with a patent foramen ovale with intermittent left to right flow, concurrent infection and malnutrition (weight 14.7 kg). This child developed a cerebral abscess and a large right fronto-parietal acute parenchymal haemorrhage whilst on anticoagulation. The mortality risk in this study is similar to the results of the review by Yang et al. [11]; a total of 122 cases of right atrial thrombosis (RAT) were identified in non-dialysis children. They showed a significant difference in the mortality for the ‘high-risk’ group (3 of 18 patients) versus ‘low-risk’ group (0 of 32 patients). In addition, mortality rate associated with CRAT is reported to be up to 18% in the adult population [6]. The largest case series (71 adult cases) showed pulmonary embolism and sepsis to be the commonest causes of death [9].

### Surveillance

CRAT is frequently asymptomatic. Currently, there is no consensus on management of asymptomatic CRAT. Anticoagulation and catheter manipulation/replacement can both result in significant adverse events as discussed. As echocardiography is already recommended for cardiovascular surveillance for children on HD, this modality may be used to screen for presence of CRAT. However, the management of incidentally diagnosed CRAT needs to be on a case-by-case basis with detailed multidisciplinary discussion.

## Conclusion

This is the first report of CRAT in a paediatric HD population. There was ~ 11% incidence of CRAT in patients receiving chronic HD at our dedicated children’s HD unit detected by surveillance echocardiography. Although frequently asymptomatic, CRAT is associated with serious sequelae. Overall, patients with CRAT were younger at the time of commencement of HD, had significantly more frequent change of CVC and received HD for a longer period of time. Anticoagulation and surveillance with expert echocardiography remain the mainstays of management. Resolution of CRAT occurs in the majority of patients. However, the condition may recur and treating CRAT may lead to adverse outcomes. Further work is required to risk stratify patients with CRAT to identify those who may benefit from treatment. This would also allow further refinement of the proposed algorithm and treatment protocols. Promotion of AVF and PD in preference to CVC-based dialysis should be encouraged to avoid this potentially serious complication.

## Electronic supplementary material

ESM 1 (PPTX 44 kb)
