# Supplementary material for: Silent and dangerous: catheter-associated right atrial thrombus (CRAT) in children on chronic haemodialysis
Source: Pediatr Nephrol. 2020 Oct 30;36(5):1245–54. doi: 10.1007/s00467-020-04743-9 (PMC8009777; doi:10.1007/s00467-020-04743-9)
Supplement: Supplementary file 1 — (PPTX 44 kb) [file 467_2020_4743_MOESM1_ESM.pptx]

## Slide 1
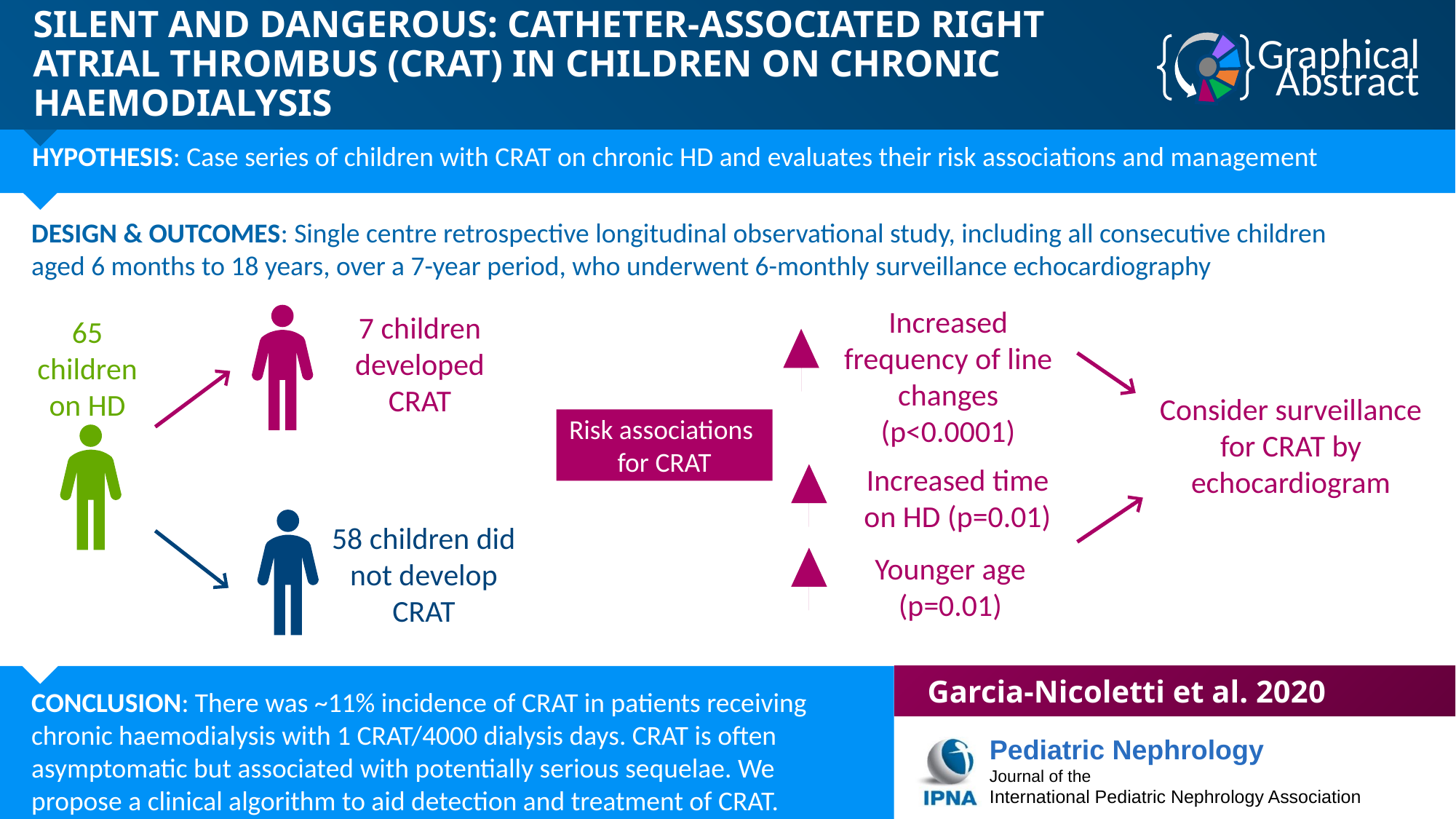

SILENT AND DANGEROUS: CATHETER-ASSOCIATED RIGHT ATRIAL THROMBUS (CRAT) IN CHILDREN ON CHRONIC HAEMODIALYSIS
HYPOTHESIS: Case series of children with CRAT on chronic HD and evaluates their risk associations and management
DESIGN & OUTCOMES: Single centre retrospective longitudinal observational study, including all consecutive children aged 6 months to 18 years, over a 7-year period, who underwent 6-monthly surveillance echocardiography
Increased frequency of line changes (p<0.0001)
7 children developed CRAT
65 children on HD
Consider surveillance for CRAT by echocardiogram
Risk associations for CRAT
Increased time on HD (p=0.01)
58 children did not develop CRAT
Younger age (p=0.01)
Garcia-Nicoletti et al. 2020
CONCLUSION: There was ~11% incidence of CRAT in patients receiving chronic haemodialysis with 1 CRAT/4000 dialysis days. CRAT is often asymptomatic but associated with potentially serious sequelae. We propose a clinical algorithm to aid detection and treatment of CRAT.
